# Proximate, minerals, carotenoid and trypsin inhibitor composition in the exoskeletons of seafood gastropods and their potentials for sustainable circular utilisation

**DOI:** 10.1038/s41598-023-38345-w

**Published:** 2023-08-11

**Authors:** Isa Olalekan Elegbede, Aderonke Lawal-Are, Rashidat Oloyede, Rukayat Oluwatayo Sanni, Toheeb Lekan Jolaosho, Appolinaire Goussanou, Vanessa Maxemilie Ngo-Massou

**Affiliations:** 1 Department of Environmental Planning, Brandenburg University of Technology (BTU), Senftenberg, Germany; 2 Department of Marine Sciences, University of Lagos, Akoka, Lagos Nigeria; 3 Department of Botany, University of Lagos, Akoka, Lagos Nigeria; 4 Department of Fisheries, Lagos State University, Ojo, Lagos Nigeria; 5 Laboratory of Animal Biotechnology and Meat Technology, University of Abomey-Calavi, Abomey-Calavi, Benin; 6 Higher Teacher’s Training College, University of Yaounde, Yaoundé, Cameroon

**Keywords:** Mass spectrometry, Marine biology

## Abstract

Periwinkle shells of *Tympanotonus fuscatus*, *Pachymelania aurita*, and *Thais coronata* were analyzed for their proximate composition, nutritionally significant minerals, trypsin inhibitors, and carotenoids. The mean values obtained were compared using an ANOVA test*.* The results showed that *T. fuscatus* had the highest mean moisture content of 0.96 ± 0.14% and a mean value of 0.49 ± 0.13% for crude fibre but was not significantly different (P > 0.05) from *P. auritus*. The crude protein and fibre content of *T. fuscatus* was significantly higher (P < 0.05) than other periwinkle samples. *T. coronata* had the highest mean total ash content and was significantly different (p < 0.05) from other periwinkle samples. *T. fuscatus* had the highest mean value for Mg (0.32 ± 0.03 mg/kg) and differed significantly (P < 0.05). The mean Ca content of *P. aurita* was not significantly different (P > 0.05) from that of *T. coronata*. The mean values of CaCO_3_ in *T. fuscatus, P. aurita, and T. coronata* were 57.20 ± 2.46, 59.50 ± 3.23, and 62.36 ± 1.56 (mg/kg), respectively. *T. coronata* was significantly different (P < 0.05) from other periwinkle samples. The mean values of carotenoids in *T. fuscatus*, *P. aurita*, and *T. coronata* were 7.17 ± 2.14, 18.00 ± 5.27, and 11.20 ± 3.60 (mg/kg), respectively, and *P. aurita* was significantly different (P < 0.05) from other periwinkle samples. *T. fuscatus* and *P. aurita* had shells with significant amounts of trypsin inhibitor (23.30 ± 4.50 mg/kg and 22.90 ± 14.10 mg/kg, respectively), making them less suitable for livestock feed. In contrast, *T. coronata* had a lower mean value of 11.80 ± 7.19 mg/kg for trypsin inhibitor, making it an excellent addition to livestock feed. The low crude fibre and fat contents of the periwinkle samples in this study make them suitable for processing complementary foods, especially for hypertensive patients. The high percentage of CaCO_3_ in periwinkle shells makes them a probable source used in the production of slurry for chromatography. The findings suggest that periwinkle shells contain specific minerals that can be applied in numerous industries. Increased use of these gastropod shells will result in successful application in product creation and a sustainable bio-circular economy.

## Introduction

Freshwater mollusc are classified into gastropods (snails, periwinkles, freshwater limpets) and bivalves (oysters, clams, mussels) with two shells that have repeatedly established a colony in the freshwater aquatic ecosystem. Gastropods have only one shell (univalves), but the shape varies among major groups^1–3^. They occur in freshwater and brackish ecosystems and are widely used as food in many countries. Periwinkles have a calcareous shell ranging in size from 10 to 30 mm. The shell is coiled in a series of whorls, which increase in diameter around the central region known as the columella, and it has a large oval opening where the body whorl terminates^4^. They are a relatively cheap source of protein, and their shells can be used in animal feed^5–7^. Periwinkles are considered one of the most crucial shellfish resources in the world. In West Africa, periwinkles have been the most dominant species among aquatic mollusc^8^. The Nigeria’s most critical mollusc shells include those of gastropods (land and water snails, periwinkles) and clams. Many publications are available on the nutritional qualities of Nigerian snails, which are in the same class as the Nigerian periwinkle. Periwinkles are rich in minerals, essential amino acids and some vitamins^9,10^. However, scanty information can only be found on the nutritional qualities and industrial importance of Nigerian periwinkles^9^, especially the shell (exoskeletal parts).

Periwinkles, which include *Tympanotonus fuscatus* and *Pachymelania aurita,* and rock snails such as *Thais coronata* belong to a large taxonomic class of invertebrates of a particular phylum of mollusca called gastropoda. Mollusc have joined or single-entity soft bodies that are covered with calcareous shells^11^. They are found basically within the littoral region of the sea, brackish or estuarine water, that are seasonal submerge regions like the mangrove swamps^12^. Although .^12^ reported that periwinkles have successfully invaded several parts of aquatic ecosystems, which is the major reason they now occupy several parts of aquatic environments, In Nigeria and other African coastal states, they are found in Lagoons, estuaries, and mangrove swamps, represented by *Pachymelania aurita* and *Tympanotonus fuscatus*^12^. These shellfish move mostly when covered by tide; however, they often remain static in a specific position once the tide is over^13^. *Tympanotonus fuscatus* and *Pachymelania aurita* are the two major species commonly found in the Niger Delta coastal region of Nigeria^14^. Similarly, *Thais coronata* (Rock snail) is a species of sea snail, specifically a marine gastropod mollusc that belongs to the family muricidae^15,16^. *T. coronata* often inhabits mangrove areas, sandy beaches, and muddy sandy substrate areas^15^. *T. coronata* is highly rich in essential fatty acids, proteins, iron, selenium, iodine, vitamin A, vitamin D, vitamin E, vitamin B6, and vitamin B12^17^.

In some parts of Nigeria, specifically the coastal areas such as Cross River and Port Harcourt, Periwinkle, especially *T. fuscatus,* commands an extremely high market value^18^. Awareness of the nutritional composition, accessibility, and low cost of periwinkle meat is the major reason for high market demand. Both periwinkle and rock snail also command high market values in Akwa Ibom State, Nigeria^19^. The activities surrounding the shellfish value chain, which includes collection, marketing, and processing, form an important economic activity among the people living in the coastal region^20^.

High demand and consumption of periwinkles in the Niger Delta area of Nigeria led to the research conducted by Job and Ekanem^21^ on the nutritional composition of two periwinkle species (*Tympanotonus fuscatus* and *Pachymelania aurita*) from a tropical creek in Nigeria. Job and Ekanem^21^ affirmed that these periwinkles contain essential nutrients required in the human body for growth and developmen*t.*

Generally, shellfish contain low cholesterol and lipids^22^, but they remain an essential source of protein, vital minerals, and some important antioxidants^21,23^. Traditional delicacies of the Efik and Ibibio ethnic groups such as "edikang ikong", "ekpang nkukwo", "afia efere," and "afang" soup, among others, are mostly prepared with periwinkle meat*.* Due to their nutritional importance and availability year-round, rich and poor people ingest them. Several low-income earners depend on shellfish meat as their primary source of protein^24^.

The fish industry cannot afford to ignore periwinkle's enormous commercial and industrial importance^25^. Periwinkle shell is domestically used as livestock feed, can be painted with various colours, and is used as an ornament for decoration^9^. Research has shown that some farmers burn shells to reduce crushing strength before grinding them for livestock feed because shells serve as a source of Ca and other mineral elements. Mollusc shells have also been a good source of lime for acidic soils^26^. Knowledge of the periwinkle shell’s biochemical composition is critical since its nutritive value is reflected in its biochemical contents^27^.

Although periwinkle and rock snails are known for their immense nutritional benefits and are heavily consumed by the Nigerian populace daily, it is worth noting that knowledge of their potential for a sustainable circular economy, especially in the context of their industrial importance, is still fragmentary. This situation has led to a low processing and utilization of these species for the development and/or production of industrial products^19^. It becomes imperative to ascertain the nutritional potential and industrial importance of periwinkle for sustainable circular utilization. This study evaluates the proximate composition, nutritionally significant minerals, trypsin inhibitor, and carotenoid contents of three species of periwinkles from the phylum *Mollusc*a, class Gastropoda: *Tympanotonus fuscatus*; *Pachymelina aurita*; and *Thais coronata,* with a view to ascertain their importance for sustainable biocircular enonomy.

## Materials and methods

### Study area

**Figure 1:**
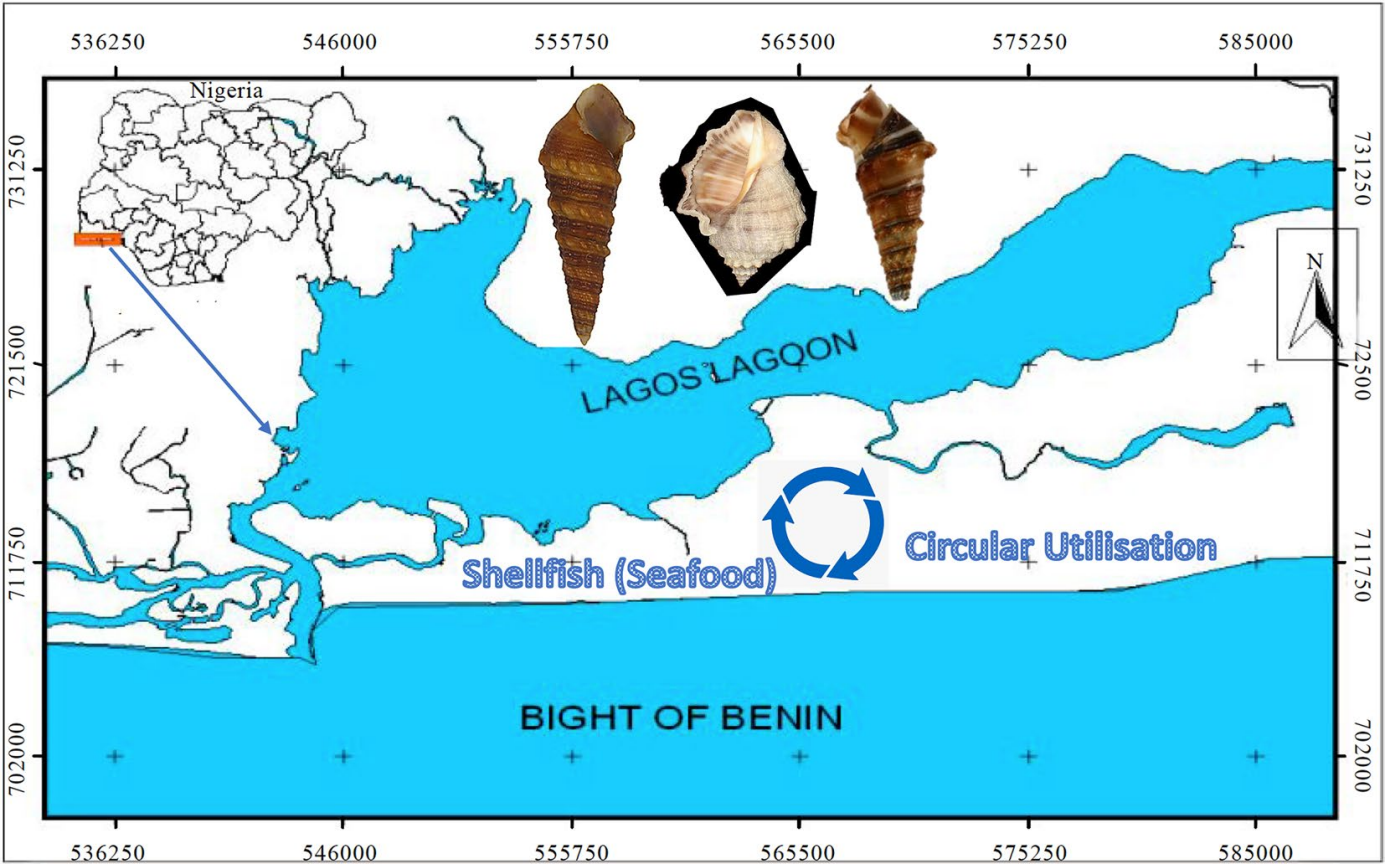
Map of Lagos Lagoon (Source:^31^).

The gastropod samples used for this study were bought from retailers who collected them from the Lagos Lagoon complex. On the West African coast, the Lagos Lagoon is the most extensive Lagoon system in the Gulf of Guinea (Fig. 1). It runs 257 km from Cotonou, Benin, to the Niger Delta, Nigeria. The Lagos Lagoon lies between longitudes 3°22′E′ and 3°4′E′ and latitudes 6°17′N′ and 6°28′N′^28^. Except for the channels, which are occasionally dredged, the Lagoon is shallow, with an average depth of 1.5 m^29^. It opens into the Gulf of Guinea through Lagos Harbor, which serves as the major extrance to the only seaport for Nigeria's western Lagoons. The sediments in Lagoons include mud, muddy sand, and sand^30^. The two main elements that govern the physicochemical parameters and biology of the Lagoon area are seawater associated with a semidiurnal tidal regime and land drainage from surrounding wetlands. Lagos Lagoon encounters brackish conditions due to seasonal fluctuations in freshwater drainage from land and seawater invasion, particularly noticeable during the dry season.

### Sample collection and description

30 samples of *Tympanotonus fuscatus*, *Pachymelania aurita,* and *Thais coronata* were collected monthly for three months (March–May in 2021 to reflect the end of the dry season and the beginning of the wet season) using a fish net. The samples were stored in a cooler that contains ice blocks to avoid decomposition during transportation. Afterwards, these species were identified based on species identification sheets from the Food and Agriculture Organization^32^, prepared by Fisher and Bianchi. *T. fuscatus* is known as periwinkle and has a single species but comprises two varieties. *Tympanotonus fuscatus* var. *fuscatus* and *Tympanotonus fuscatus* var radula inhabit quiet waters and muddy areas, and the substratum is rich in decaying organic matter. *T. fuscatus* var. *fuscatus* is differentiated by the presence of turreted, granular and spiny shells with tapering ends. In contrast, *T. fuscatus* var. *radula* is characterized by the absence of tiny tubercles on the shell, differentiating it from the other variety. They primarily feed on algae and diatoms. *T. fuscatus* possesses an elongated shell with a regular, increasing whorl; the ribs are weakly V curved with much fine striation, and blackish brown stripes are visible on the shell^8,33^. *P. aurita* is morphologically different from *T. fuscatus*. The shell of *P. aurita* is characterized by sharp spines, and it has a broader aperture; In *P. aurita*, the sharpness of the spine solidly depends on the organism's age; as the organism becomes older, the spine of the shell becomes blunt and thicker, and it is one of the most common *mollusc*s in the Lagos Lagoon. The genus Pachymelina has been said to be endemic to West Africa^34,35^. *Thais coronata* is also a univalve gastropod. It can be found in estuaries, brackish water creeks, and mangrove swamps in many freshwater bodies across Nigeria. The length of the shells was adequately accessed and measured. The edible parts were carefully removed from the shells with a drilling pin before washing them in distilled water to remove dirt and debris before measurement and drying. After that, the samples were sent to the University of Ibadan, Oyo State Laboratory for analysis.

### Proximate analysis

#### Determination of crude fat content using the soxhlet extraction method

Well-blended samples (5.00 g) were weighed into the thimbles and placed with cotton wool to prevent the sample from pouring out during extraction. The round bottom flask was dried in an oven at 60 °C, and the initial empty weight was recorded. 80 ml of hexane was poured into the flask, and the thimble containing the sample was fitted/placed into the extractor. The heating mantle was switched on, and water was set to run through the condenser for cooling. The extraction was allowed to continue its reflux for 2 h after it was discontinued. The flask was then dried in the oven to eliminate all hexane. The amount of crude fat or oil (%) present in the sample was calculated by subtracting the weight of the empty flask from the final weight*.*

1 $${\text{Fat}}\;{\text{content}}\;\left( \% \right) = \frac{{{\text{weight}}\;{\text{of}}\;{\text{flask}}\;{\text{after}}\;{\text{extraction}}\;\& \;{\text{drying}} - {\text{the}}\;{\text{weight}}\;{\text{of}}\;{\text{empty}}\;{\text{flask}}}}{{{\text{Sample}}\;{\text{weight}}}} \times 100$$

### Determination of ash

Empty crucibles were dried in an oven at 130 °C for 30 min to remove the moisture present on the crucibles, transferred into a desiccator and allowed to cool at room temperature for approximately 20 min. Then, the weight of the empty crucibles was taken and recorded as W_0_. The samples were then blended into powder using a mortar and pestle to increase the surface area. We weighed 1.0000 g of the sample using an analytical balance into the crucibles and recorded it as W_1_, then ashed in the furnace at 500 ± 15 °C for 5–6 h^36^. The crucibles containing the samples were allowed to cool for approximately 30 min in the furnace. Then, with the crucible tongue, the crucibles were transferred into the desiccator and allowed to cool at room temperature for approximately 45 min. The final weight of the crucible and content was then taken and recorded as W_2._

2 $$\mathrm{Ash} \; \mathrm{content }\;(\mathrm{\%})=\frac{\mathrm{W}_{2}-\mathrm{W}_{0}}{\mathrm{W}_{1}} \times 100$$

W_0_: weight of empty crucible, W_1_: weight of crucible containing samples, W_2_: weight of crucible containing ashed content.

### Determination of protein content according to the Kjeldahl method

A total of 1.00 g of well-prepared sample was weighed to an accuracy of 0.1 mg into a 250 ml digestion tube, and 2kjeltabs Cu 3.5 (alternatively 7 g K_2_SO_4_ and 0.8 g CuSO_4_⋅5H_2_0) from Finlab Nigeria limited was added. Then, 12 ml of concentrated H_2_SO_4_ was carefully added and gently shaken to wet the sample with acid. The exhaust system was then added to the digestion tubes in the rack, and the water aspirator was set to full effect*.* The samples were digested for 1 h at 420 °C. The racks of tubes were removed, placed on a stand and allowed to cool for 10–20 min. The tubes were then inserted into the distillation unit, and the safety door closed; 80 ml of deionized water was carefully added to the tubes. Then, 25–30 ml receiver solution was added to the conical flask and placed into the distillation unit*.* The platform was placed, so the distillate outlet was submerged in the receiver solution.

50 ml of 40% NaOH was dispensed into the tube and distilled for approximately 5 min. Then, the distillate was titrated with standardized 35% HCl of 0.1 ml until the blue‒gray endpoint was achieved. A blank was run through all the steps above.

3 $${\text{Crude}}\;{\text{Protein}}\;\left( \% \right) = \frac{{\left( {{\text{T}} - {\text{B}}} \right) \times {\text{N}} \times 14.007 \times 100}}{{{\text{W}}_{1}\;\left( {{\text{mg}}} \right)}} \times {\text{F}}$$

W_1_ = sample weight (mg), T = titration volume of sample (ml), B = titration volume of blank (ml), N = normality of acid to 4 decimal places, F = conversion factor for nitrogen to protein = 6.25 for food and feed.

### Determination of fibre using fibretec

Samples (2.0 g) were digested in 20 ml of 1.25% H_2_SO_4_, and the mixture was boiled for 30 min and then filtered and washed with hot water to reduce the acidity. This process was tested with pH paper; the residue was again digested in 20 ml of 1.25% NAOH^37^. The mixture was heated for 30 min, filtered and washed with hot water, dried in an oven, transferred to a platinum crucible, weighed (W_1_), heated in a furnace at 550 °C and weighed again (W_2_).

4 $$\mathrm{Percentage \; crude \; fibre }\;(\mathrm{\%})=\frac{\mathrm{Weight \;of \;oven}-\mathrm{dried\; sample}-\mathrm{Weight \;of \;burnt \;sample }}{\mathrm{Weight \;of \;sample\; used}} \times 100$$

### Determination of moisture content

Empty porcelain crucibles were dried in an oven at 105 ± 50 °C for 30 min to remove moisture from the dishes. Then, the porcelain crucibles were transferred into a desiccator and allowed to cool at room temperature for 20 min; the weight of the empty porcelain crucibles was taken and recorded as W_0._ The samples were blended into powder using a mortar and pestle to increase the surface area; 1.00 g of sample was weighed into porcelain crucibles (recorded as W_1_) and dried in an oven at 105 ± 50 °C for 4 h. The porcelain crucibles containing the samples were allowed to cool for approximately 10 min in the oven with the use of a crucible tongue. The porcelain crucibles were transferred into a desiccator and allowed to cool at room temperature for approximately 30 min. The final weight of the porcelain crucibles and content was recorded as W_2_.

5 $$\mathrm{Moisture\; content }\;(\mathrm{\%})=\frac{\left(\mathrm{W}_{0}+\mathrm{W}_{1}\right)-(\mathrm{W}_{0}+\mathrm{W}_{2})}{\mathrm{W}_{1}} \times 100$$

### Nitrogen free extract (N.F.E)

The N.F.E. which is the non-evaluated proximate composition parameters such as digestible carbohydrate and soluble organic compounds, was determined by subtracting the sum (%) of all other evaluated proximate composition parameters from 100. The equation below was adopted^38^.

6 $${\text{N}}.{\text{F}}.{\text{E }}\left( \% \right) \, = { 1}00 \, {-} \, \left( {{\text{P1 }} + {\text{ P2 }} + {\text{ P3 }} + {\text{P4 }} + {\text{P5}}} \right)$$

where: P1 = moisture content (%), P2 = crude protein content (%), P3 = crude fat content (%), P4 = crude fibre content (%), P5 = ash content (%).

### Determination of minerals

The nitric-perchloric acid digestion method is followed to determine Ca, K, Mg, P, and CaCO_3_ from the periwinkle shell. For this digestion, 1.00 g of periwinkle shell sample was weighed into a digestion tube or a conical flask, and 10 ml of H_2_SO_4_ and 30 ml of nitric acid were added. It was placed on a hot plate in a fume cupboard and digested until the digest became clear and then diluted to 100 ml, after which it was taken to AAS for mineral determination. According to the method^37^, the absorbance was determined by atomic absorption spectroscopy (AAS) (UV visible U-2900 Hitachi, Tokyo, Japan) at wavelengths of 285.2 nm (Mg), 766.5 nm (K), 422.7 nm (Ca), 213.62 nm (P) and 2155.12 nm (CaCO_3_). AAS standard solutions (1000 mg/l in 5% HNO_3_) (Merck, Germany) were used for calibration. Finally, lanthanum and cesium chloride (0.1%) were added to the samples and standards to mitigate interferences. The AAS siphoning hose was then placed into the digested sample. After running, the standards for the mineral/metal were determined, and the concentration of the mineral/metals in the solution displayed on the computer screen was read.

### Determination of carotenoid content (mg/kg)

100 mg of sample was weighed into a centrifuge tube, and 10 ml of 80% acetone was added, appropriately mixed and centrifuged at 3000 rpm for 10 min and filtered. The supernatant was brought up to a volume of 10 ml using 80% acetone according to a previously described method^39^. Then, the optical density (OD) (absorbance) was read at a wavelength of 450 nm using spectrophotometer (UV visible U-2900 Hitachi, Tokyo, Japan).

Note: Similar AAS machine was used for absorbance determination of mineral parameters and carotenoid but was adjusted at different wavelengths. The reason is because the UV 2900 version can be used for multifunctional analyses.

7 $${\text{Total}}\;{\text{carotenoid}}\;{\text{content }}\left( {{\text{mg}}/{\text{kg}}} \right) = \frac{{{4 } \times {\text{ OD }} \times {\text{ total vol}}.{\text{ of sample }} \times { 1}000}}{{\text{Sample weight}}}$$

### Determination of trypsin inhibitor

1 g of properly blended died sample was measured into a flask, and 50 ml of 0.5 M NaCl was added. The mixture was quivered for about 30 min and centrifuged at 1500 rpm for 5 min. 10 ml of decanted filtrate was pipetted into another flask; then, 2 ml of standard trypsin solution of 2 mg/l was added to it*.* The absorbance was caliberated at 410 nm while 10 ml of the filtired sample was used as blank. Standard trypsin inhibitors (1, 2, 4, 6, 8, and 10 mg/l) were prepared with N-alpha-benzoyl-dl-arginine-p-nitroanilide (BAPNA), and their absorbencies were measured at 410 nm. An accurate graph which illustrated the absorbance against the concentration was drawn and extrapolated, by tracing the absorbance to the concentration axis and the trypsin inhibitor content was obtained.

8 $${\text{Trypsin inhibitor }}\left( {{\text{mg}}/{\text{kg}}} \right) = \frac{{{\text{Conc}}.{\text{ obtained in mg}}/{\text{l }} \times {\text{ volume of sample }} \times {\text{ DF}}}}{{\text{Sample weight}}}$$

DF: Dilution factor. If not diluted, then DF = 1.

### Statistical analysis

Microsoft Excel version 16.0 was used to evaluate the mean ± standard deviation of the raw data and descriptive computation. One way Analysis of variance (ANOVA) at significance level at 5% (P < 0.05) and the correlation coefficients were done using SPSS IBM, 15.0. The disparity of mean between samples was achieved through Duncan multiple post HOC test.

## Results and discussion

The obtained laboratory results for the proximate analysis, mineral nutrients and phytochemical components are shown graphically (Figs. 2, 3, 4, 5, 6, 7, 8, 9, 10).

**Figure 2:**
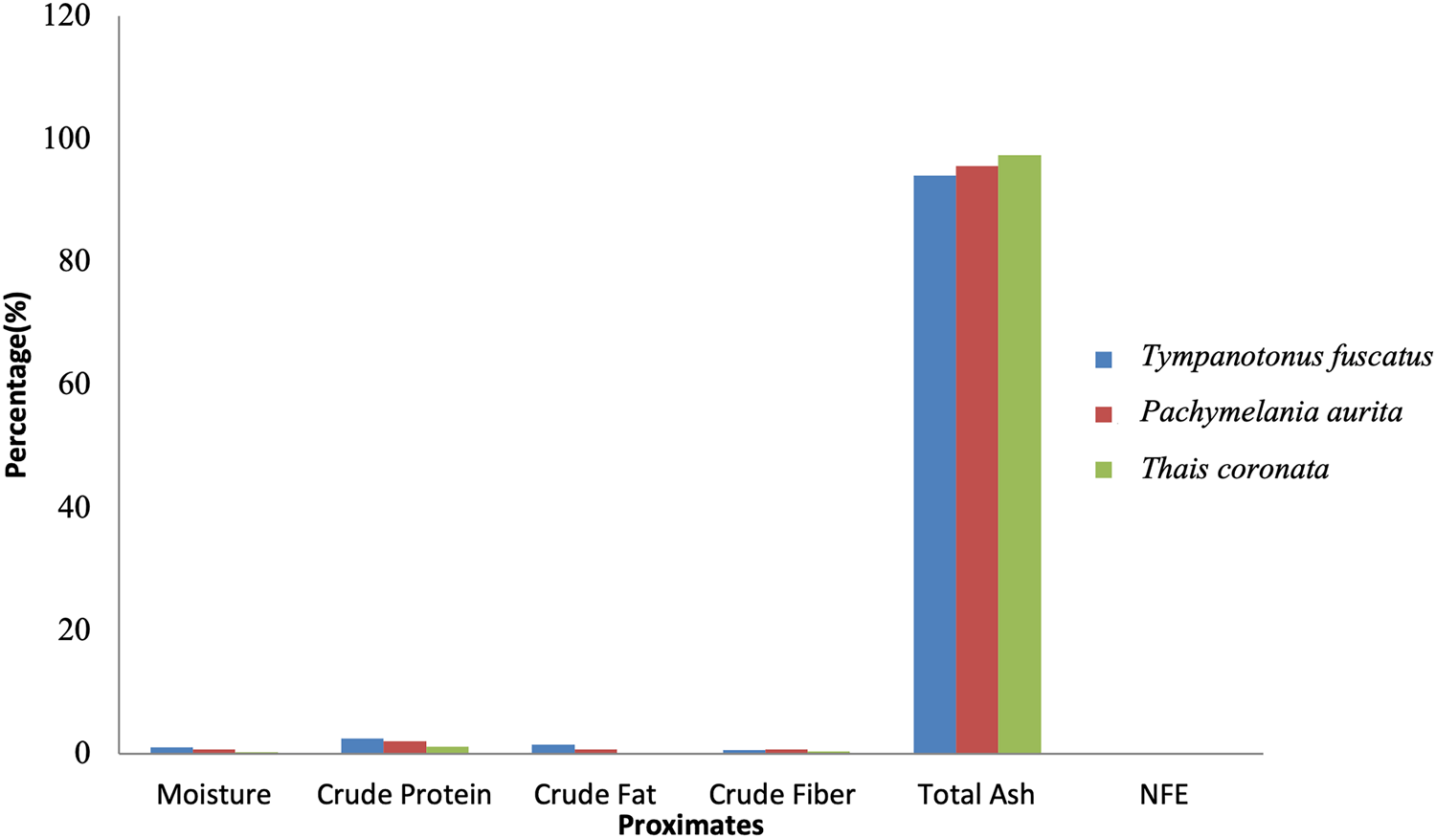
Variations in the proximate composition of *Tympanotonus fuscatus*, *Pachymelania aurita* and *Thais coronata* in March.

**Figure 3:**
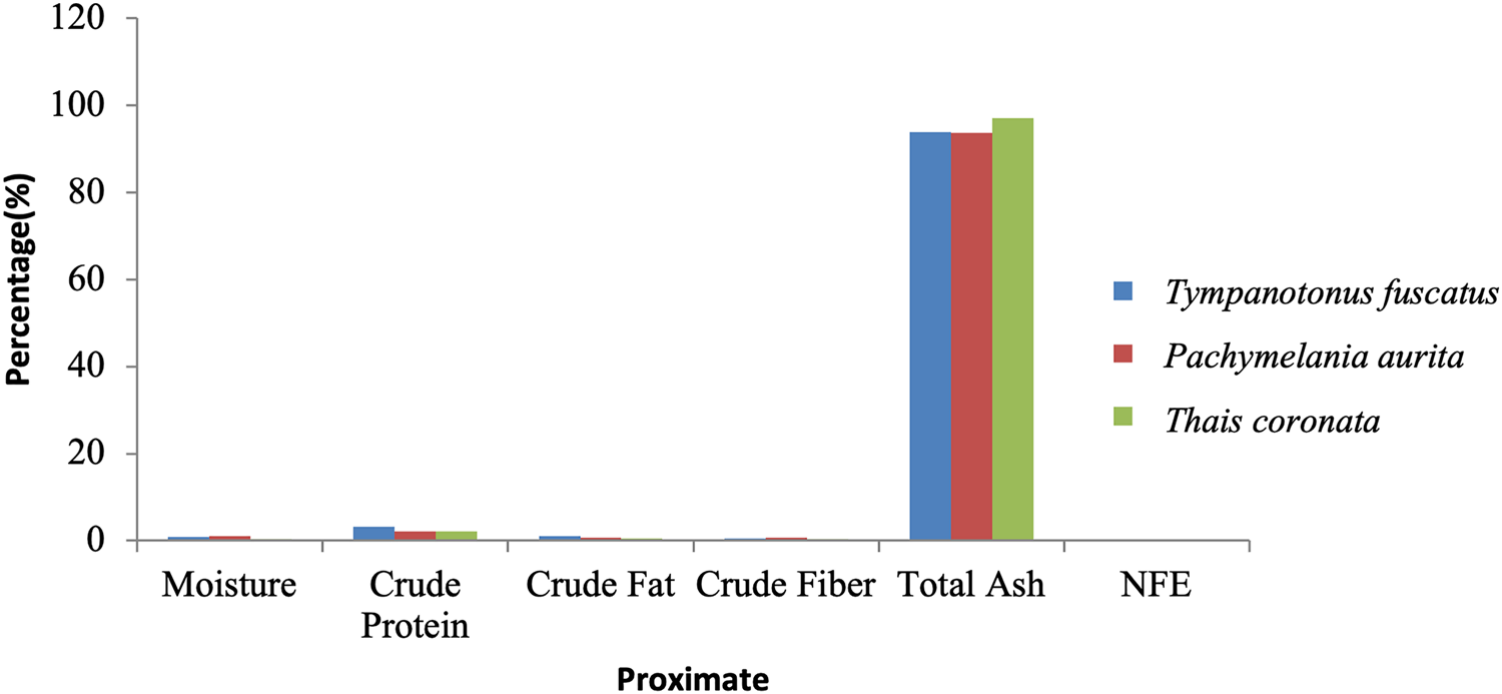
Variations in the proximate composition of *Tympanotonus fuscatus*, *Pachymelania aurita* and *Thais coronata* in April.

**Figure 4:**
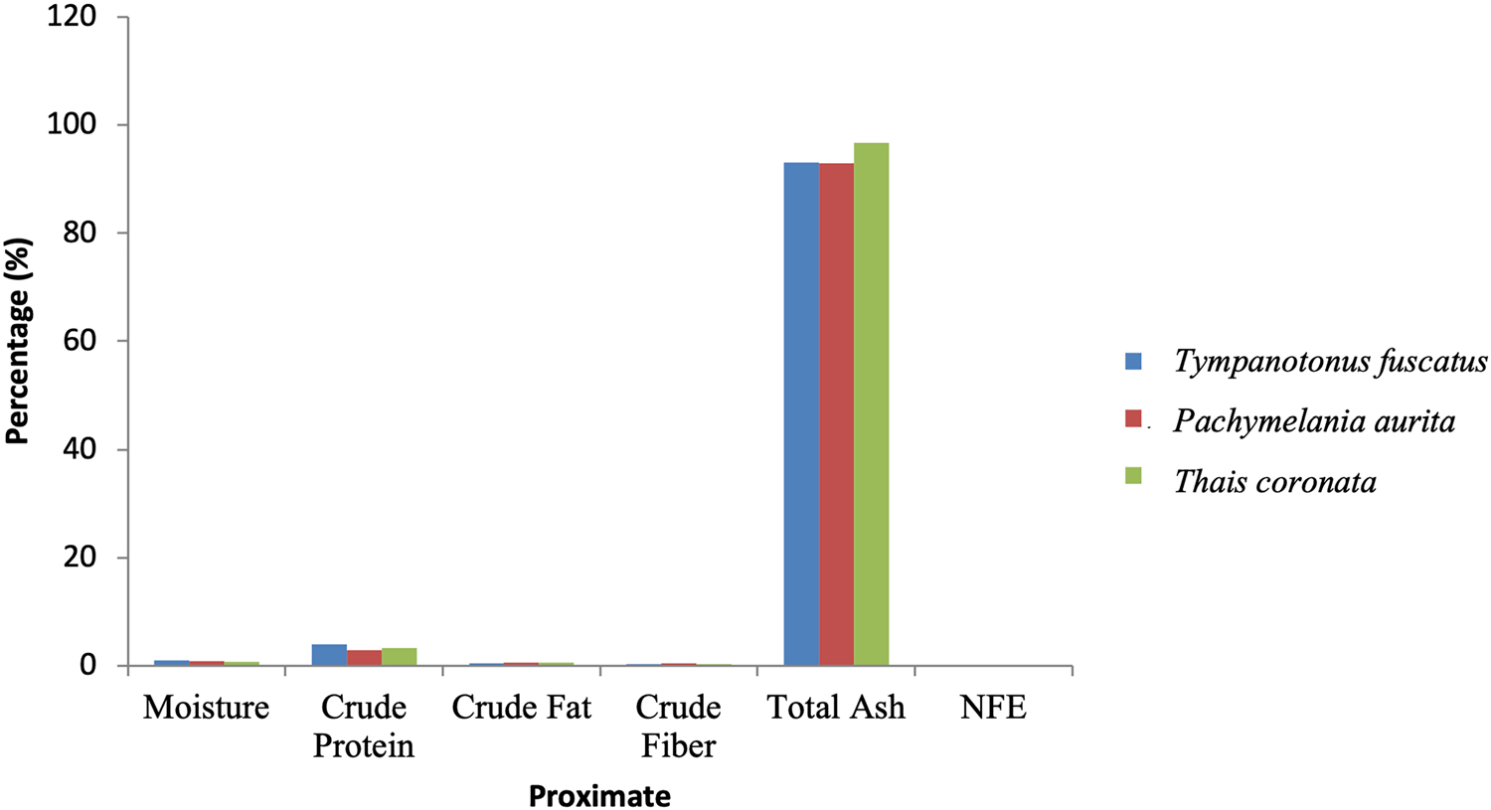
Variations in the proximate composition of *Tympanotonus fuscatus*, *Pachymelania aurita* and *Thais coronata* in May.

**Figure 5:**
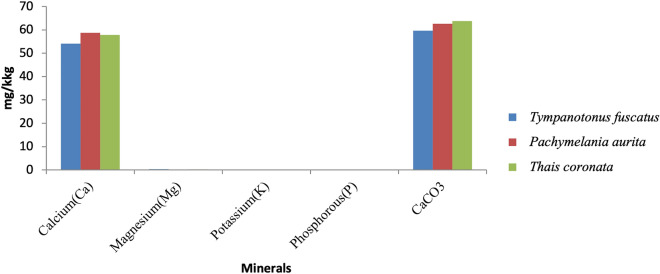
Variations in the Minerals of *Tympanotonus fuscatus*, *Pachymelania aurita* and *Thais coronata* in March.

**Figure 6:**
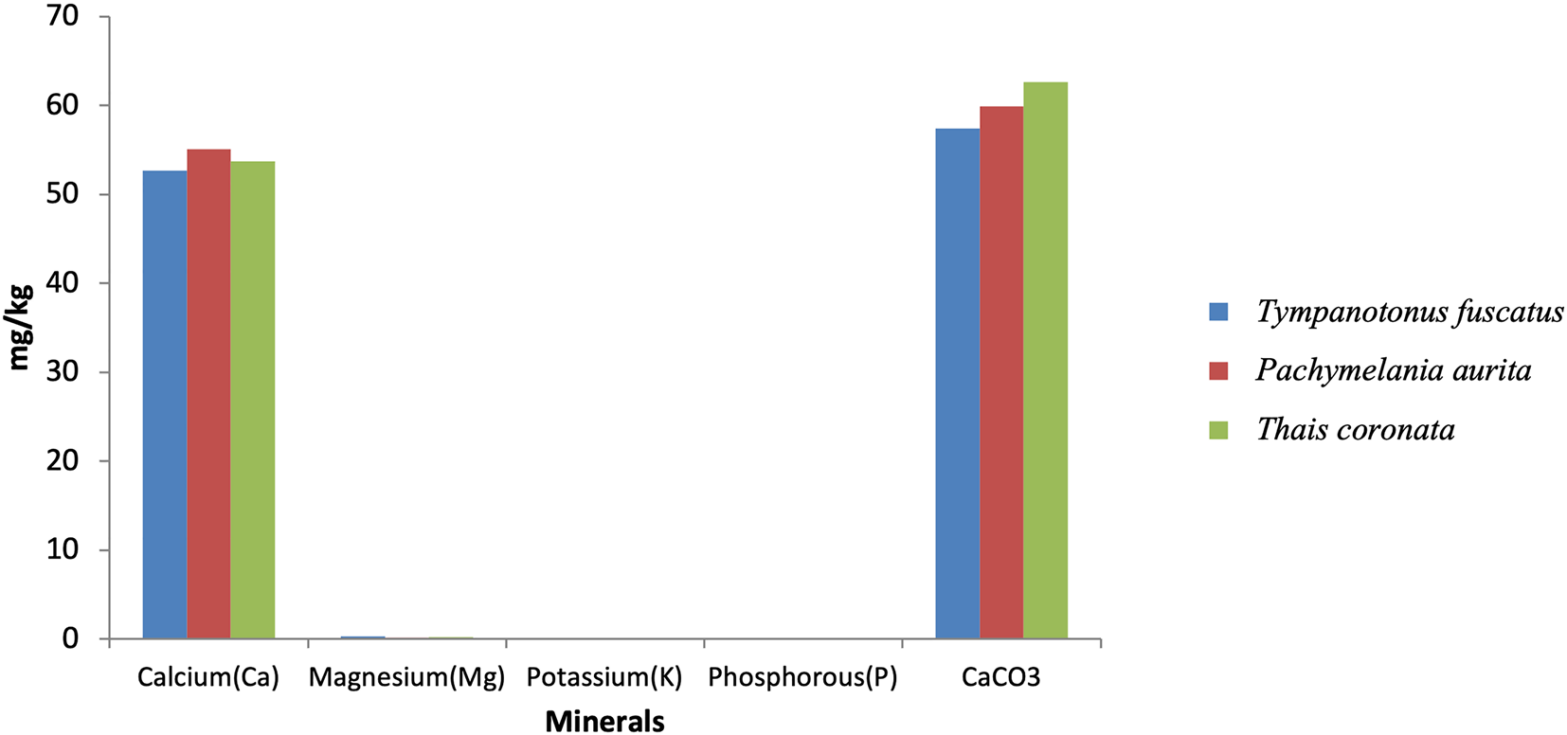
Variations in the Minerals of *Tympanotonus fuscatus*, *Pachymelania aurita* and *Thais coronata* in April.

**Figure 7:**
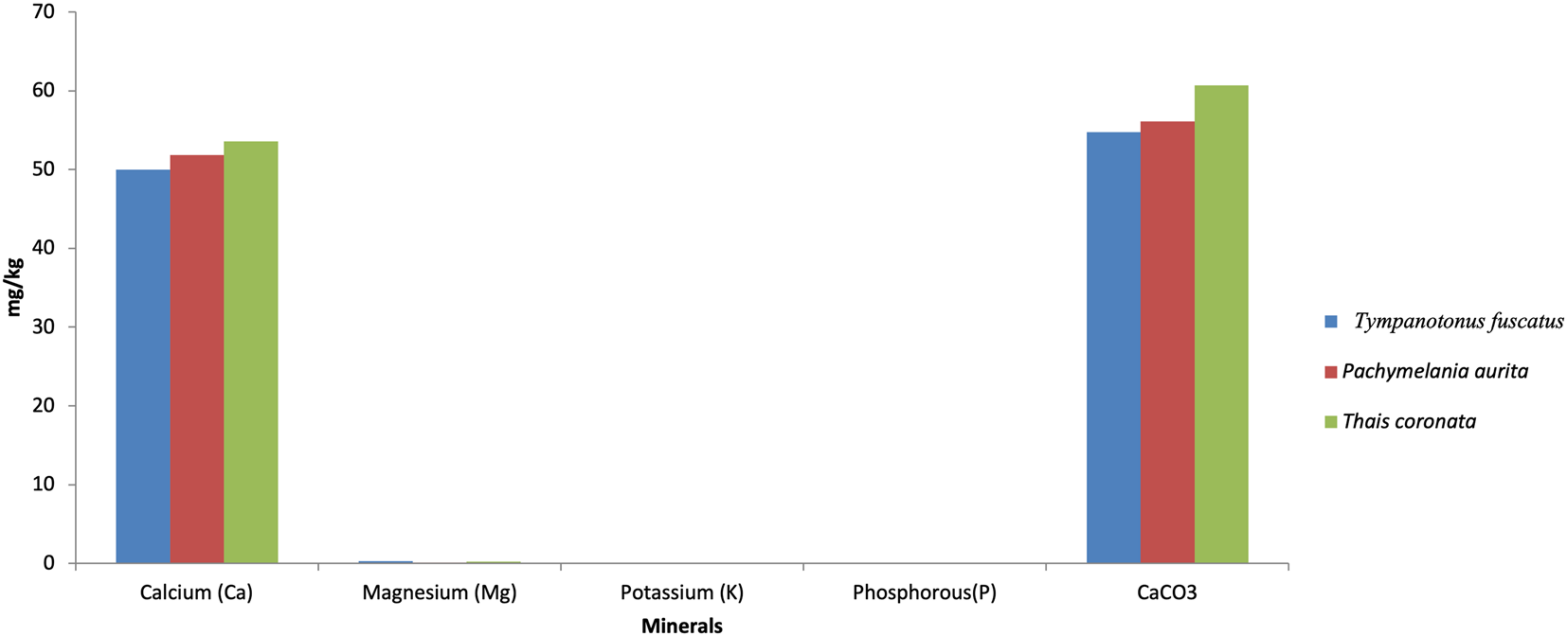
Variations in the Minerals of *Tympanotonus fuscatus*, *Pachymelania aurita* and *Thais coronata* in May.

**Figure 8:**
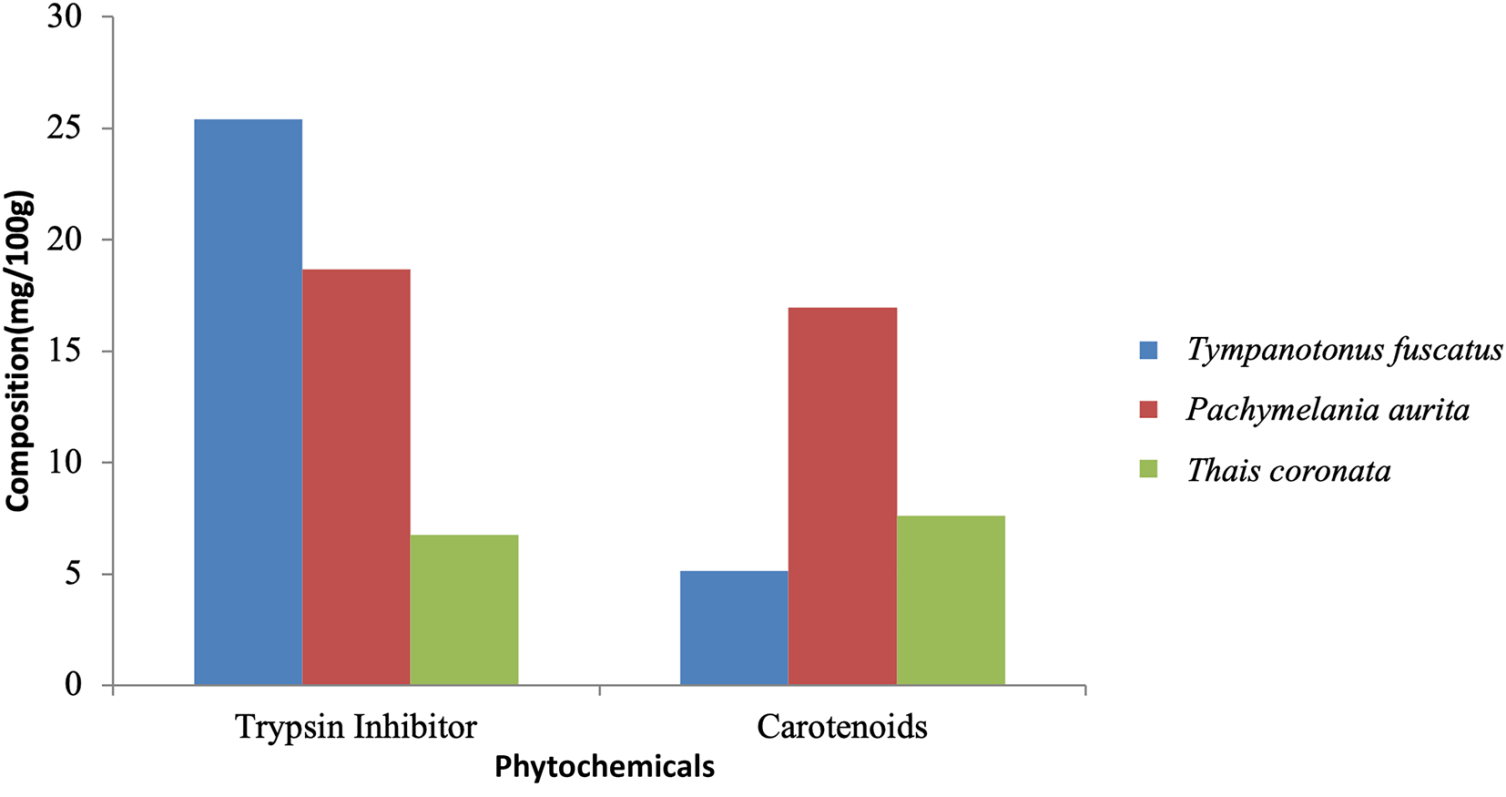
Variations in the phytochemical contents of *Tympanotonus fuscatus*, *Pachymelania aurita* and *Thais coronata* in March.

**Figure 9:**
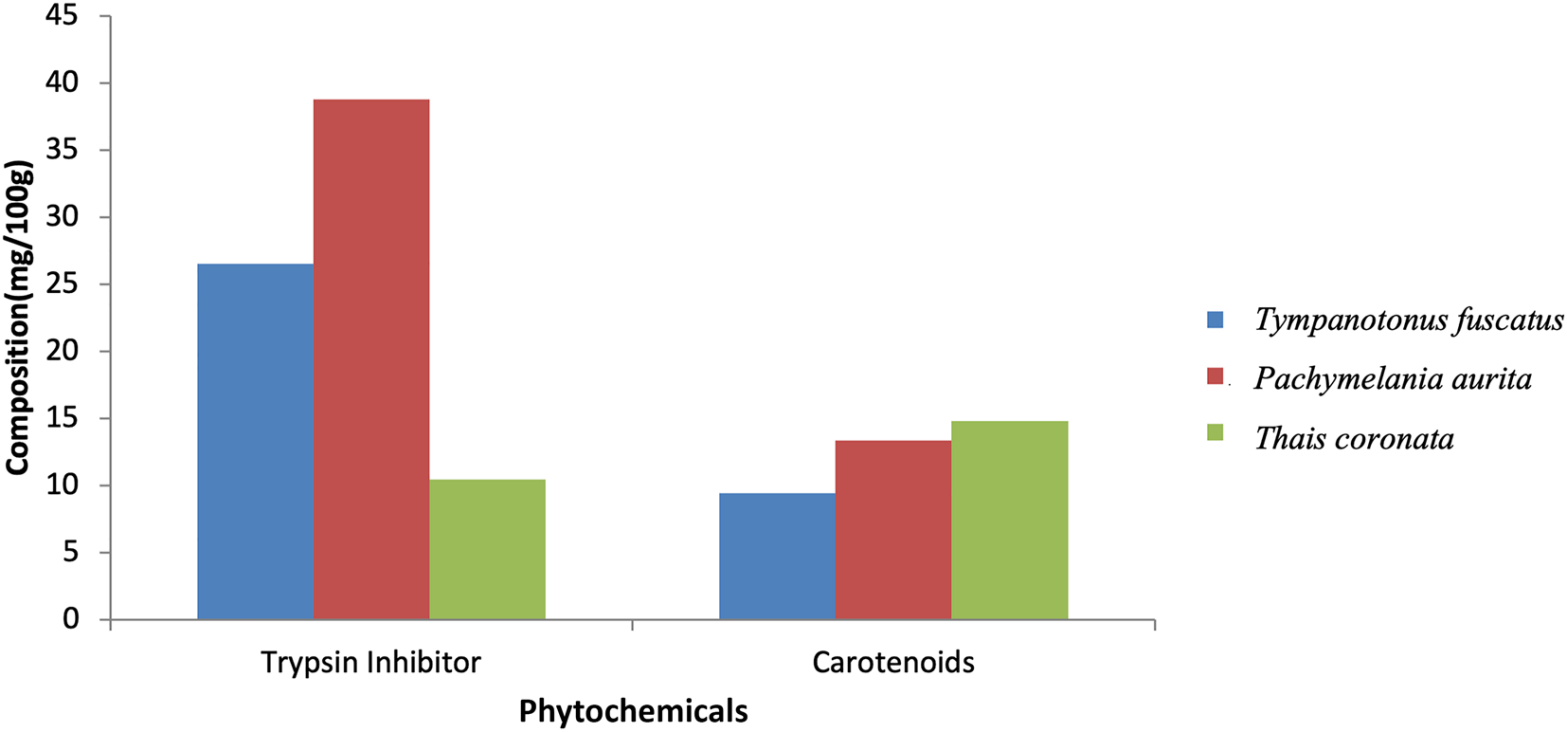
Variations in the phytochemical contents of *Tympanotonus fuscatus*, *Pachymelania aurita* and *Thais coronata* in April.

**Figure 10:**
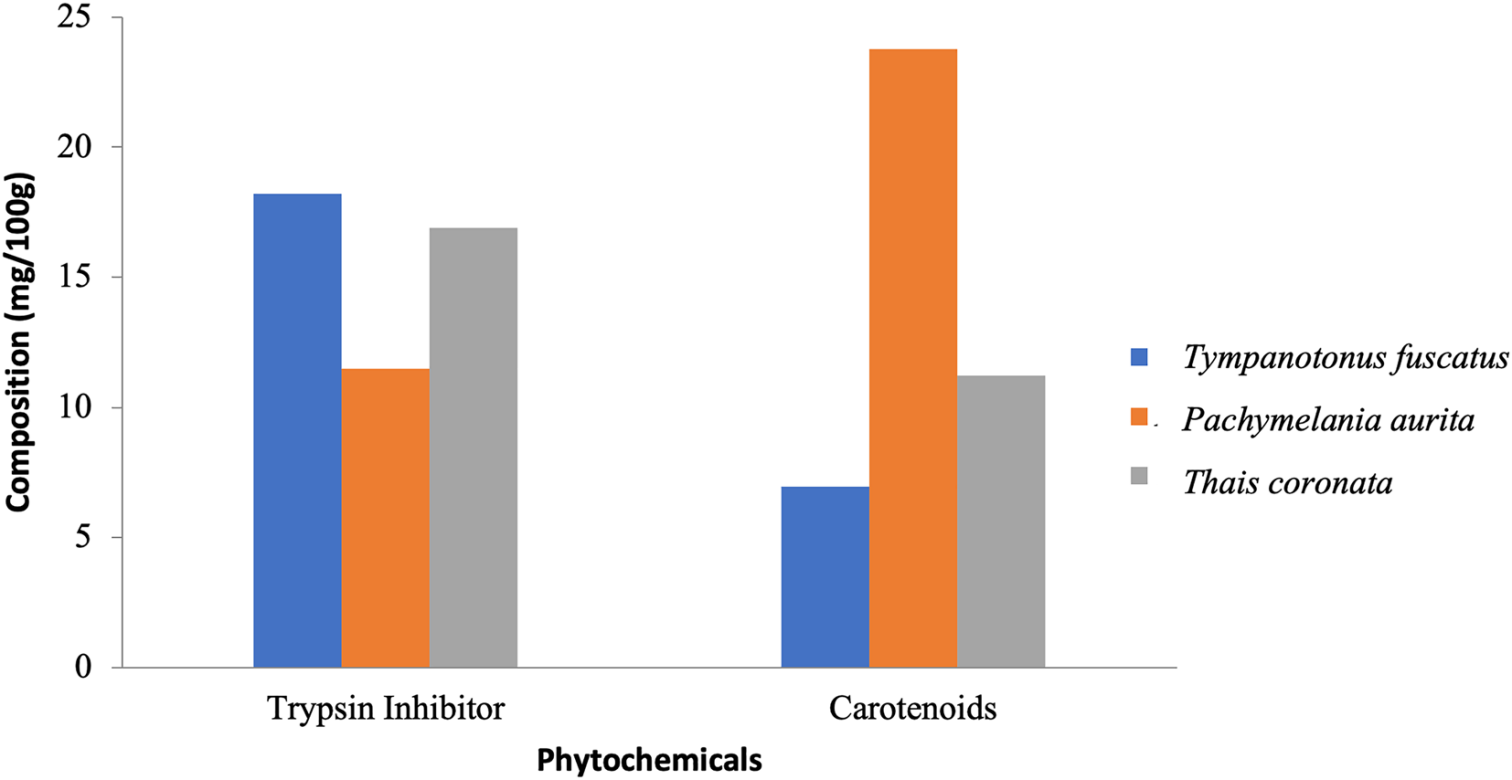
Variations in the phytochemical contents of *Tympanotonus fuscatus*, *Pachymelania aurita* and *Thais coronata* in May.

Figure 2 shows a graphical representation of the sample’s proximate constituents in March. The amount of moisture was highest in *T. fuscatus* at 1.09% and lowest in *T. coronata* at 0.3%. *T. fuscatus* was also found to be the highest in crude protein, while *T. coronata* was found to be the lowest in crude protein and crude fat at 1.18% and 0%, respectively, while *T. fuscatus* was the highest in crude fat at 1.50%. *P. aurita* has the highest crude fibre content at 0.74%, and *T. coronata* has the lowest at 0.38%; for the ash content, *T. coronata* was found to have the highest content at 97.38% and the lowest *T. fuscatus* at 94.08%, while the nitrogen-free extract was highest in *T. coronata* at 0.76% and lowest in *P. aurita*.

Figure 3 depicts the proximate contents of the samples in April. The moisture content of *P. aurita* had the highest value at 0.99%, with *T. coronata* having the lowest value at 0.40% (Fig. 3). *T. fuscatus* was also found to have the maximum protein and fat at 3.21% and 1.08%, respectively, while *T. coronata* was also the lowest in crude protein and crude fat at 2.00% and 0.50%, respectively. *P. aurita* has the highest value of crude fibre at 0.61% and nitrogen-free extract at 1.75%, while *T. fuscatus* has the lowest ash content at 93.89%.

Figure 4 revealed the sample’s proximate composition in the month of May. *T. fuscatus* had the highest moisture and crude fibre values at 0.99% and 3.95%, while *P. aurita* had the highest values in crude fat and crude fibre at 0.60% and 0.48%, respectively. *T. coronata* had the highest ash content at 96.74% and *T. fuscatus* the lowest at 93.12%, while *T. coronata* had a negative value in the nitrogen-free extract at -1.71%.

The proximate analysis results expressed as mean value and standard deviation were found to be significantly different as shown in Table 1. Basically, the nutritional constituents of finfish and shellfish vary, due to differences in the type of species, individuals, part (such as tissue, shell or flesh) evaluated, sex differences, season^40^, the morphological status of such species, and the ecological and geographical structure of the ecosystem where such species were extracted. Awareness of the moisture constituent of any aquatic species is crucial because it provides valuable information on the quality and vulnerability of organisms to microbial spoilage and fungal infection^40,41^. It helps stabilize the movement of organisms. Organisms with low moisture content are less susceptible to microbial spoilage^42,43^ and have the ability to prolong their shelf life and retain their nutritive content for a considerable period of time during postharvest*. T. fuscatus* had the highest mean moisture content at 0.96 ± 0.14% and was significantly different (P < 0.05) from other samples, while *T. coronata* had the lowest mean moisture level at 0.46 ± 0.20%. The present research does not agree with the report of Oyawoye et al.^44^ whose work was on *T. fuscatus* shell from Nembe, Rivers’ state. The results contradict the values obtained from this study, such that the moisture content value obtained in the periwinkle shell was 2.5%, compared to 0.96 ± 0.14% in this study. The result obtained for moisture is considerably similar to that obtained in the research carried out by Etim^26^; Omotoso^45^; and Elegbede et al.^43^.

**Table 1:** Mean value of proximate analysis (%) of the sample periwinkles.

Parameters (%)	*T. fuscatus*	*P. aurita*	*T.* coronata
Moisture	0.96 ± 0.14^a^	0.89 ± 0.09^a^	0.46 ± 0.20^b^
Crude protein	3.23 ± 0.70^a^	2.37 ± 0.44^b^	2.18 ± 1.06^c^
Crude fat	1.02 ± 0.50^a^	0.69 ± 0.09^b^	0.36 ± 0.32^c^
Crude fibre	0.49 ± 0.13^a^	0.61 ± 0.13^a^	0.36 ± 0.01^b^
Total ash	93.60 ± 0.51^b^	94.00 ± 1.35^c^	97.00 ± 0.32^a^
NFE	0.59 ± 0.47^b^	1.33 ± 1.14^a^	0.43 ± 1.23^c^

Values are given as the mean ± standard deviation of the samples analyzed in monthly triplicates using ANOVA. In each row, the mean with common superscripts is not significantly different.

Generally, the types of proteins present in shellfish are basically rich and provide essential amino acids needed for body growth, reproductive processes, and antioxidant synthesis, particularly in living organisms^46^. This agrees with the assertion of Okuzumi and Fujii^47^ that proteins are large biomolecules that are naturally significant and beneficial to living organisms and are needed for sustenance. Protein constituents are found abundantly in several shellfishes, such as oysters and bivalves, compared to finfishes^43,48^. The presence of proteins in the human body helps to repair damaged tissues and other important biochemical and anatomical^49–51^. The highest percentage of mean crude protein was found in *T. fuscatus* at 3.23 ± 0.70% and showed a significant difference (P < 0.05), and the lowest percentage of crude protein was found in *T. coronata* at 2.18 ± 1.06%. Akpan and Oscar^18^ recorded a protein content of 22.52% for *T. fuscatus* from the Calabar River, which was proportionally higher than the 3.23% obtained in this study. Similarly, the result obtained for protein content in this study was lower than the values of 46.51%, 51.30%, and 58.45% recorded in blended marrows of *T. fuscatus*, *P. aurita*, and *T. coronata*, respectively, by Inyang et al.^19^. A high protein content of 49.54% by Adebayo-Tayo et al.^12^ and 48.62% by Job and Ekanem^21^ was recorded in the marrow of *P. aurita*.

Just like protein, fat is also an essential molecule that helps in the body's skeletal formation, allows free movements and distributions of soluble vitamins, aids the retention of protein from food^43^, and is involved in cell biological processes in the human body. The fat content recorded in this study ranged from 0.36 to 1.02%, and a significant difference (P < 0.05) exists. *P. aurita* was found to have the highest level of crude fibre at 0.61 ± 0.13% but showed no significant difference (P > 0.05) to *T. fuscatus*, and the lowest level of crude fibre was found in *T. coronata* at 0.36 ± 0.01%. The mean value for crude fibre in *T. fuscatus* (0.49%) corresponds with the 0.043% registered by Oyawoye et al.^44^ for similar species. The fat contents recorded in samples of this study were relatively lower than those by Inyang et al.^19^ for three shellfishes and also lower compared to those obtained in similar species by Job and Ekanem^21^, and Ehigiator and Oterai^52^. Differences in several proximate constituents of the species in our study and those obtained by previous researchers could be attributed to the exoskeletons evaluated in this study compared to marrow or meat evaluated by previous researchers^43^. The low-fat level of these exoskeletons means they could be processed into powders and subsequently added to foods as a supplement for people who are hypertensive and individuals who are suffering from lipid-related diseases such as arteriosclerosis^19,43^.

The total ash, which serves as a signal that indicates the mineral availability in any organism, also helps in proper body formation^43,51^. High total ash contents in periwinkle exoskeletons (*T. coronata* at 97.0 ± 0.32% was the highest and the lowest in *T. fuscatus* at 93.6 ± 0.51%) mean the presence of macro-minerals, which include P, Mg, Ca, and their derivative compounds^53^. Although our result was higher than those recorded by^12,19,21,52^. The extremely high amount of total ash in all periwinkle shell samples could be due to chitin availability in shells, enhanced by Ca and its derivative^54^, or by the presence of high inorganic adulterants or activated carbon obtained following calcination.

Low crude fibre was obtained in all samples, ranging from 0.36% in *T. coronata* to 0.49% in *T. fuscatus* at P < 0.05. Adebayo-Tayo et al.^55^ and Lah et al.^56^ also observed variations in crude fat in shellfish. The results of this study were similar to those obtained by^19,21,52^ for similar species. Inyang et al.^19^ affirmed that the low crude fibre in periwinkle makes it suitable for processing complementary foods.

High levels of NFE were recorded in *P. aurita* at 1.33 ± 1.14%, and the least NFE was registered in *T. coronata* at 0.43 ± 1.23%. *P. aurita* showed significant differences (P < 0.05) from other samples.

Correlation matrixes between the proximate components for *T. fuscatus*, *P. aurita,* and *T. coronata* were recorded (Tables 2 and 3). There was a robust positive correlation between the nitrogen-free extract and crude protein and a strong negative correlation between the nitrogen-free extract and crude fat (Table 2).

**Table 2:** Correlation coefficient of the proximate composition in *T. fuscatus.*

Parameters	Moisture	C.Pro	C.Fat	C.Fibre	Total ash	NFE
Moisture %	1					
Crude protein	− 0.308	1				
Crude fat	0.252	− 0.998	1			
Crude fibre	0.570	− 0.957	0.939	1		
Total ash	0.007	− 0.953	0.970	0.825	1	
NFL	− 0.293	0.999	− 0.999	− 0.953	− 0.958	1

*C.Pro:* crude protein, *C.Fat:* crude fat, *C.Fibre:* crude fibre, *NFE:* nitrogen free extract.

**Table 3:** Correlation coefficient of the proximate composition in *P. aurita*.

Parameters	Moisture	C.Protein	C.Fat	C.Fibre	Total ash	N.F.E
Moisture %	1					
Crude protein	0.176	1				
Crude fat	− 0.5	− 0.940	1			
Crude fibre	− 0.526	− 0.929	0.999	1		
Total ash	− 0.689	− 0.835	0.972	0.979	1	
NFL	0.765	0.768	− 0.940	− 0.950	− 0.994	1

*C.Pro:* crude protein, *C.Fat:* crude fat, *C.Fibre:* crude fibre, *NFE:* nitrogen free extract.

A robust positive correlation was observed between crude fibre and crude fat, and a strong negative correlation was observed between nitrogen-free extract and total ash (Table 3).

There was a strong positive correlation between the nitrogen-free extract and total ash and a strong negative correlation between the nitrogen-free extract and crude protein (Table 4).

**Table 4:** Correlation coefficient of proximate composition in *T. coronata* .

Parameters	Moisture	C.Pro	C.Fat	C.Fibre	Total ash	NFE
Moisture %	1					
Crude protein	0.983	1				
Crude fat	0.797	0.895	1			
Crude fibre	− 0.693	− 0.815	− 0.988	1		
Total ash	− 0.938	− 0.986	− 0.957	0.900	1	
NFL	− 0.975	− 0.999	− 0.911	0.835	0.992	1

*C.Pro:* crude protein, *C.Fat:* crude fat, *C.Fibre:* crude fibre, *NFE:* nitrogen free extract.

Figure 5 depicts the mineral contents of the exoskeleton samples in March. The mineral composition of the samples for March shows that *P. aurita* was found to be higher in Ca at 58.78 mg/kg, and *T. fuscatus* was found to be higher in Mg, K, and P at 0.035 mg/kg, 0.022 mg/kg, and 0.71 mg/kg, respectively, while CaCO_3_ was found to be highest in *T. coronata* at 63.75 mg/kg.

Figure 6 shows the mineral constituents of the samples in April. The mineral composition of the samples recorded Ca to be higher in *P. aurita* in April at 55.10%. *T. fuscatus* was the lowest at 53.72 mg/kg. *T. fuscatus* was found to have the maximum Mg, K and P at 0.31 mg/kg, 0.017 mg/kg, and 0.065 mg/kg, respectively, while *T. coronata* was found to have the highest CaCO_3_ at 62.65 mg/kg.

Figure 7 illustrates the mineral contents of the samples in May. The mineral composition of the samples for May shows *T. coronata* to have the highest Ca contents of 52.58 mg/kg, while *T. fuscatus* has the highest values of Mg, K, and P at 0.296 mg/kg, 0.014 mg/kg, and 0.058 mg/kg, respectively (Fig. 7). *T. coronata* was recorded to have the highest CaCO_3_ content with 60.67 mg/kg.

The phytochemical composition of the samples in March showed that *T. fuscatus* had the highest trypsin inhibitor content at 25.39 mg/kg, *while P. aurita* had the highest carotenoid content at 16.87 mg/kg (Fig. 8). The phytochemical composition of the samples in April showed that *P. aurita* had the highest trypsin inhibitor content at 38.77 mg/kg, *T. coronata* had the highest carotenoid content at 14.81 mg/kg, and *T. fuscatus* had the highest carotenoid content at 9.43 mg/kg (Fig. 9). The phytochemical composition of the samples for May shows that *T. fuscatus* had the highest value of trypsin inhibitor at 18.21 mg/kg, while *P. aurita* had the highest value of carotenoids at 23.76 mg/kg (Fig. 10).

The mean level of minerals in exoskeletons was presented, and a significant difference (P < 0.05) was also established (Table 5). The major mineral in natural periwinkle shells as obtained in our study is basically CaCO_3_, with mean contents of 57.20 ± 2.46 mg/kg, 59.50 ± 3.23 mg/kg, and 62.36 ± 1.56 mg/kg in *T. fuscatus*, *P. aurita,* and *T. coronata,* respectively, as shown in Table 5. The obtained results are in consonance with the typical mineral compositions registered by Orji et al.^57^ in their findings. Periwinkle shell grains comprising mineral components of over 85% are sufficient for the production of sandpaper abrasives^58^. Having a higher percentage of Ca composition, Oyawoye et al.^44^ also suggested that the shells will be suitable for use in soda ash production, sugar refining, and flue gas desulfurization due to the high demand for limestone. It’s so surprising that the major macrominerals believed to generally enhance the morphological development of calcareous exoskeletons were obtained in extremely high amounts, which contradicts the assertion of Davies and Jamabo^59^ that shells generally contain macrominerals in extremely high quantities. Na and its derivative element NaCO_3_ are another mineral whose absence was not expected since they both play good roles in ensuring the compactness of shells and help stabilize the pH required during the development of precipites^43^. The absence of Na and its derivative element NaCO3 corroborates the finding of Elegbede et al.^43^.

**Table 5:** Mean value of mineral composition (mg/kg) of the periwinkle samples.

Parameters (mg/kg)	*T. fuscatus*	*P. aurita*	*T. coronata*
Ca	52.20 ± 2.08^b^	55.20 ± 3.44^a^	55.00 ± 2.40^a^
Mg	0.32 ± 0.03^a^	0.12 ± 0.004^c^	0.20 ± 0.01^b^
k	0.02 ± 0.00^b^	0.02 ± 0.00^b^	0.14 ± 0.001^a^
P	0.06 ± 0.01^a^	0.05 ± 0.01^a^	0.02 ± 0.00^b^
Trypsin inhibitor	23.30 ± 4.50^a^	22.90 ± 14.10^b^	11.80 ± 7.19^b^
Carotenoids	7.17 ± 2.14^c^	18.00 ± 5.27^a^	11.20 ± 3.60^b^
CaCO_3_	57.20 ± 2.46^b^	59.50 ± 3.23^b^	62.36 ± 1.56^a^

Values are given as the mean ± standard deviation of the samples analyzed in monthly triplicates using ANOVA. In each row, the mean with common superscripts is not significantly different.

The highest mean value for Ca was found in *P. aurita* at 55.20 ± 3.44 mg/kg. *T. fuscatus* had the highest mean levels of Mg, K, and P (0.32 ± 0.03 mg/kg, 0.017 ± 0.004 mg/kg, and 0.06 ± 0.006 mg/kg, respectively). Trypsin inhibitor was found to be high in *T. fuscatus* at 23.3 ± 4.50 mg/kg, while carotenoids were lowest in *T. coronata* at 11.2 ± 3.60 mg/kg_._ The CaCO_3_ content ranged from 57.2 to 62.36 mg/kg, and the mean highest Ca content was found in *T. coronata* (Table 5). Due to the high content of CaCO_3_ in periwinkle shells, previous research^43^ has reported that the periwinkle shell could be applied in designs, the pearl button industry, and the construction industry. Shells and gravel are used for building roads, and lime from the shell is a vital component in the production of concrete and plaster^60^. In this study, the mean value of CaCO_3_ in the shells of *T. fuscatus*, *P. aurita*, and *T. coronata* was 57.2 mg/kg, 59.5 mg/kg, and 62.36 mg/kg, respectively, making *T. coronata* the species with the highest CaCO_3_ content at 62.36 mg/kg. The report of Orji et al.^61^ on the valorization of shells based on mineral components denotes a possible utilization potential of periwinkle shells as adsorbent materials in production thin layer chromatography plates. The high percentage of CaCO_3_ in periwinkle shells makes them a probable source of CaCO_3_, demonstrating that the powdered shells of *T. fuscatus* and *L*. *littorea* may be used in producing slurry for thin-layer chromatography. *T. fuscatus* in this study has a high amount of CaO compared to work done by Malu and Bassey^62^. CaCO_3_ in periwinkles can also serve as supplements added to the cultivation medium to derive the optimal pH that is favorable for the fungus's growth^60^. Shells of periwinkle can also be a suitable replacement for asbestos in brake pad production^58^. Azor et al.^63^ illustrated the potential of melon and snail shell waste mixtures as a supplementary source of carbohydrates that enhance the compactness and hardness of mild steel. Periwinkle (*T. fuscatus*) shells can serve as an alternative source of lime for the glass industry^62^. Ademola et al.^64^ asserted that periwinkle shell with a high mineral component can be valorized and used as a supplementary material in the production of cooking utensils; it can be utilized directly for the treatment of measles; and it can also be used as a bioindicator of toxic metals in the aquatic ecosystem^65^. The high availability of Ca and its derivative compound (CaCO_3_) makes the periwinkle shells of this study important to the biomedical industry in that they can be utilized as an alternative to porcelain used for the production of dental implant materials such as artificial teeth^43,51^, which will not only reduce the expenses involved but also help to alleviate the environmental concern associated with the indiscriminate dumping of these shells. The essentiality of Ca in human nutrition and body functions cannot be overemphasized. Ca could be obtained from both animal and plant sources, and typical examples include dairy products such as milk, broccoli, e.t*.*c^43^. Elegbede et al.^43^ also mentioned the significance of bivalve shells in agriculture, such that blended shells could be incorporated into animal-formulated diets. Scientific trials on the successful utilization of shells in several applications, particularly cosmetics, have been conducted, although none of these have yielded any positive outcomes. However, Azor et al.^66^ investigated the suitability of periwinkle shells as carburizing materials for the surface hardness improvement of low-carbon steel. Shell waste has been successfully tested as a suppressant of specific toxic metals present in wastewater and other industrial effluents^67–70^. Oyster shell powder can supplement the sawdust medium to increase the calcium content of mushrooms.

The mean content of phytochemicals in the exoskeleton samples is depicted in Table 5. *T. fuscatus* had the highest trypsin inhibitor content at 23.30 ± 4.50 mg/kg, while *P. aurita* had the highest carotenoid content at 18.00 ± 5.27 mg/kg (Table 5). Carotenoids are phytochemicals naturally occurring in plants, vegetables, fruits, and seeds. Some carotenoids are also synthesized artificially for food coloring, such as b-carotene^71^. Carotenoids can be found in various plants, such as citrus fruits, soybean meal, and vegetables, including green leafy vegetables, spinach, pumpkins, and potatoes, among others^72^. Carotenoids in the human diet help lower chronic illnesses connected to coronary heart disease (CHD) and help prevent bronchial and uterine-related issues and prostate and colon cancers. Trypsin inhibitors are phytochemicals naturally found in legumes, soybeans, and other natural products. Trypsin inhibitors are anti-nutritional factors that inhibit the digestion of protein when consumed, resulting in growth deficiency in livestock^73^. No research has been conducted on the presence of trypsin inhibitors in the periwinkle shell in the Lagos Lagoon. Generally, animals, such as mollusc, do not synthesize carotenoids de novo as well as trypsin inhibitors. However, the presence of carotenoids and trypsin inhibitors in periwinkle samples in this study might be due to the presence of these phytochemicals in the food they ingested, which subsequently bioaccumulate in their bodies. Cohen et al.^74^ affirmed that trypsin plays a major role in the decomposition of several proteins, specifically in humans, animals with only one chambered stomach, and young ruminants. Modification of metabolic processes might also be largely responsible for the presence of carotenoids and trypsin inhibitors in them^75–77^. Several studies have been conducted to investigate the presence of carotenoids in periwinkle shells^72^. One such study is the report of Withnall et al.^78^, on Raman spectra of carotenoids in natural products, where carotenoids were discovered in the shell of the periwinkle *Littorina littorea*. Ituen^79^ mentioned that mollusc shells are used in livestock feeding. Hence, this study investigated the presence of trypsin inhibitors in periwinkle shells and showed that the shells of *T. fuscatus* and *P. aurita* possess a considerable mean percentage of trypsin inhibitors (Table 5). These results may render them less suitable for livestock feeding. *T.* corona has a lower mean value of 11.80%, which projects it as a suitable addition to livestock feed. Nonetheless, shells are known for their hardiness and compactness attributes; this means that shells can be partially utilized as a supplementary filler in the production of materials in the cement industry, such that it helps to enhance the ability of cement to moisten and aids the hardness and compactness of the cement during mixtures^80^. Kozminsky and Lezin^81^ also researched the distribution of pigments in the gastropod Littorina obtusata from Kandalaksha Bay, the White Sea, and the results showed that varieties of carotene pigments were discovered in the shell.

The mineral composition in *T.* coronata shows a strong positive correlation between K and Mg and a strong negative correlation between the trypsin inhibitor and P (Table 8). The correlation matrixes between the mineral components of periwinkle exoskeletons are presented in Tables 6, 7, 8. A robust positive correlation was observed between CaCO_3_ and P, and a strong negative correlation was observed between carotenoids and Mg (Table 6). The mean values of mineral contents of the periwinkle (*Littorea littorina*) evaluated were 11.83 mg/kg for Mg, 0.40 mg/kg for K, and 0.92 mg/kg for *P.* Although these values are low, they are significantly higher than the mean values registered for *Pachymelania aurita*. This finding indicates that the mean value for minerals derived in this study is relatively low compared to other periwinkle shells.

**Table 6:** Correlation coefficient of mineral and phytochemical contents in *T. fuscatus*.

Parameters	Ca	Mg	K	P	*T.* inhibitor	Carotenoids	CaCO_3_
Ca	1						
Mg	0.897	1					
K	0.949	0.991	1				
P	0.991	0.948	0.982	1			
*T.* inhibitor	0.890	0.598	0.702	0.823	1		
Carotenoids	− 0.253	− 0.654	− 0.544	− 0.378	0.215	1	
CaCO_3_	0.992	0.946	0.982	0.999	0.825	− 0.375	1

*Ca:* calcium, *Mg:* magnesium, *K:* potassium, *P:* phosphorus, *T. inhibitor:* trypsin inhibitor, *CaCO*_*3*_ : calcium carbonate.

**Table 7:** Correlation coefficient of mineral composition in *P. aurita*.

Parameters	Ca	Mg	K	P	*T.* inhibitor	Carotenoids	CaCO_3_
Ca	1						
Mg	0.999	1					
K	0.996	0.999	1				
P	0.994	0.997	0.999	1			
*T.* Inhibitor	0.224	0.192	0.141	0.113	1		
Carotenoids	− 0.620	− 0.594	− 0.552	− 0.528	− 0.904	1	
CaCO_3_	0.992	0.987	0.977	0.971	0.348	− 0.716	1

Ca: calcium, Mg: magnesium, K: potassium, P: phosphorus, T. inhibitor: trypsin inhibitor, CaCO_3_: calcium carbonate.

**Table 8:** Correlation coefficient of mineral composition in *T. coronata*.

Parameters	Ca	Mg	K	P	*T.* inhibitor	Carotenoids	CaCO_3_
Ca	1						
Mg	0.959	1					
K	0.954	0.999	1				
P	0.775	0.922	0.929	1			
*T.* inhibitor	− 0.797	− 0.936	− 0.942	− 0.999	1		
Carotenoids	− 0.850	− 0.666	− 0.653	− 0.326	0.360	1	
CaCO_3_	0.792	0.933	0.939	0.999	− 0.999	− 0.353	1

*Ca:* calcium, *Mg:* magnesium, *K:* potassium, *P:* phosphorus, *T. inhibitor*: trypsin inhibitor, *CaCO*_*3*_ : calcium carbonate.

The mineral composition of *P. aurita* shows a strong positive correlation between P and K and a strong negative correlation between carotenoids and trypsin inhibitors (Table 7).

The mineral composition of *T.* coronata shows a strong positive correlation between K and Mg and a strong negative correlation between the trypsin inhibitor and P (Table 8).

## Conclusion

This study is the first documented report that will serve as a reference point for future research on the proximate composition, mineral content, and phytochemical composition of periwinkles from the Lagos Lagoon complex and their potential for a sustainable circular economy. This present study uses three commercially essential gastropod shells, *Tympanotonus fuscatus*, *Pachymelania aurita,* and *Thais coronata*, in the Lagos Lagoon. The study results showed variations among species based on their proximate, mineral, and phytochemical components. For nutritional purposes, *T. fuscatus* was the best due to its high fat and protein content and low ash and crude fibre content*.* The low crude fibre and fat content obtained in all samples makes them suitable for processing complementary foods, especially for those who are hypertensive and whose systolic and diastolic blood pressures are above 160 mmHg and 95 mmHg, respectively, and those suffering from fat-related diseases. The mineral compositions (Ca and CaCO_3_) of the three gastropods were high. This situation indicates that the Lagoon is in good shape for some mollusc in terms of food availability and abundance. They may have devised a method for dealing with and adapting to constant pollution in the environment*.* Periwinkle shells can be beneficial for various purposes, as they possess certain minerals that can be utilized in various fields. Sustainable incorporation of these shells in several applications after processing will help to enhance the usage of these essential, less expensive, and readily available materials while also promoting a circular bio-economy. Periwinkle shell applications will contribute to the valorization of the economy, create employment, and pave the way for waste management through a proper recycling system. However, an urgent examination of the Lagos Lagoon's environmental conditions is needed, as some of the samples in this study are not favorable in reference to their nutritional contents.

## Data Availability

The datasets generated and/or analyzed during the study are available from the corresponding author upon reasonable request.
